# 1-(5-Acetyl-2-hy­droxy­phen­yl)ethanone

**DOI:** 10.1107/S1600536811020423

**Published:** 2011-06-11

**Authors:** Jörg Hübscher, Lidiya Izotova, Samat Talipov, Frank Eissmann, Edwin Weber

**Affiliations:** a Institut für Organische Chemie, TU Bergakademie Freiberg, Leipziger Strasse 29, D-09596 Freiberg/Sachsen, Germany; b Institute of Bioorganic Chemistry, Academy of Sciences of Uzbekistan, M. Ulugbek Street 83, 100125 Tashkent, Uzbekistan

## Abstract

The crystal structure of the title compound, C_10_H_10_O_3_, is characterized by classical intra­molecular hydrogen bonding. The hy­droxy group is disordered over two positions (77 and 23%). The crystal structure is stabilized *via* π–π [3.5986 (1) Å] and weak nonclassical C—H⋯O inter­actions [3.2797 (15) Å].

## Related literature

For hydrogen bonding, see: Desiraju & Steiner (1999 ▶). For π–π inter­actions, see: Janiak (2000 ▶). For the anti­fungal activity of the title compound, see: Prats *et al.* (2007 ▶).
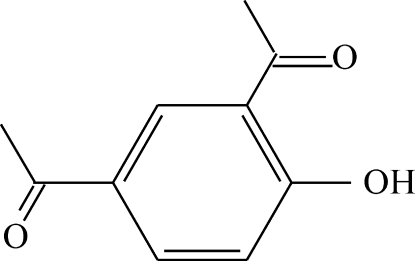


## Experimental

### 

#### Crystal data

C_10_H_10_O_3_
*M* *_r_* = 178.18
Monoclinic, 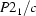

*a* = 7.1134 (2) Å
*b* = 10.9260 (3) Å
*c* = 11.5291 (3) Å
β = 98.485 (2)°
*V* = 886.25 (4) Å^3^
*Z* = 4
Mo *K*α radiation
μ = 0.10 mm^−1^
*T* = 153 K
0.60 × 0.57 × 0.52 mm

#### Data collection

Bruker Kappa APEXII CCD diffractometer
Absorption correction: multi-scan (*SADABS*; Bruker, 2007 ▶) *T*_min_ = 0.659, *T*_max_ = 0.747
9182 measured reflections
1855 independent reflections
1577 reflections with *I* > 2σ(*I*)
*R*_int_ = 0.018

#### Refinement

*R*[*F* ^2^ > 2σ(*F* ^2^)] = 0.041
*wR*(*F* ^2^) = 0.123
*S* = 1.11
1855 reflections
132 parameters
H-atom parameters constrained
Δρ_max_ = 0.30 e Å^−3^
Δρ_min_ = −0.21 e Å^−3^

### 

Data collection: *APEX2* (Bruker, 2007 ▶); cell refinement: *SAINT* (Bruker, 2007 ▶); data reduction: *SAINT*; program(s) used to solve structure: *SHELXS97* (Sheldrick, 2008 ▶); program(s) used to refine structure: *SHELXL97* (Sheldrick, 2008 ▶); molecular graphics: *SHELXTL* (Sheldrick, 2008 ▶); software used to prepare material for publication: *SHELXTL*.

## Supplementary Material

Crystal structure: contains datablock(s) I, global. DOI: 10.1107/S1600536811020423/rk2275sup1.cif

Structure factors: contains datablock(s) I. DOI: 10.1107/S1600536811020423/rk2275Isup2.hkl

Supplementary material file. DOI: 10.1107/S1600536811020423/rk2275Isup3.cml

Additional supplementary materials:  crystallographic information; 3D view; checkCIF report

## Figures and Tables

**Table 1:** Hydrogen-bond geometry (Å, °)

*D*—H⋯*A*	*D*—H	H⋯*A*	*D*⋯*A*	*D*—H⋯*A*
O1—H1O⋯O2	0.84	1.78	2.5240 (15)	147
O1*A*—H1O*A*⋯O3	0.84	1.58	2.369 (6)	156
C6—H6⋯O3^i^	0.95	2.55	3.2797 (15)	134

Symmetry code: (i) 
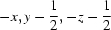
.

**Fig. 1.**
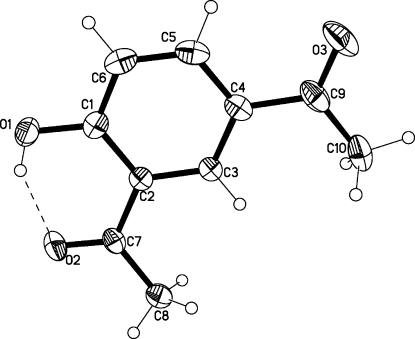
Perspective view of the title compound with the atom numbring scheme. Displacement ellipsoids are drawn at 40% probability level. Dashed line represents intramolecular H–bond. Only major moiety of disordered hydroxy–group is presented.

**Fig. 2.**
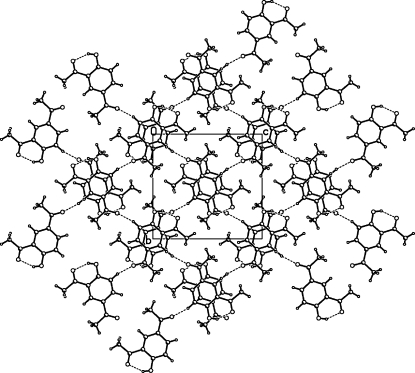
Packing diagram of the title compound, viewed down the a–axis. The weak hydrogen bonds are shown as dashed lines.

## supplementary crystallographic information

### Comment

The title compound is known for its high antifungal activity (Prats *et al.*, 2007). In the molecule, the hydroxy–group is disordered over two positions [O1—H10 and O1A—H10A with occupancies of 0.769 (3) and 0.231 (3), respectively] related to each other *via* 180° rotation about the C3–C6 molecule axis. The molecule is almost planar with a maximum derivation from the mean plane of all non hydrogen atoms (RMS = 0.0458) of 0.1185 (14)Å for the methyl atom C8 (Fig. 1). The bond lengths and bond angles of the benzene ring are normal. The carbonyl and the disordered hydroxy–group of the molecule are involved in classical intramolecular H–bonds (O1—H1O···O2, 2.5240 (15)Å, 147.3°; O1A—H1OA···O3 2.369 (6)Å, 155.8°), while no conventional intermolecular hydrogen bond is found in the packing structure. The structure is stabilized *via*π–π–interactions (Janiak, 2000) involving the neighboring diacylphenol molecules in the stacks - Cg···Cg distance between consecutive molecules is 3.5986 (1)Å, which run in direction of the crystallographic *a*–axis (Fig. 2). Interaction between these stacks is realised *via* weak C—H···O interactions (Desiraju & Steiner, 1999) C6—H6···O3^i^ (3.2797 (15)Å). Symmetry code: (i) -*x*; *y*-1/2; -*z*-1/2.

### Experimental

The title compound has been obtained as a by–product during attempted formation of a complex between 2–acetyl–4–ethynylphenol with Co(II) in *Me*OH solution due to hydrolysis. The prismatic shaped crystals are yellow colored and stable in the air.

### Refinement

H atoms were positioned geometrically and allowed to ride on their respective parent atoms, with C—H = 0.98Å and *U*_iso_(H) = 1.5*U*_eq_(C) for methyl, C—H = 0.95Å and *U*_iso_(H) = 1.2*U*_eq_(C) for aryl and O—H = 0.84Å and *U*_iso_(H) = 1.5*U*_eq_(O) for hydroxy atoms.

### Crystal data

**Table:**

C_10_H_10_O_3_	*F*(000) = 376
*M**_r_* = 178.18	*D*_x_ = 1.335 Mg m^−^^3^
Monoclinic, *P*2_1_/*c*	Mo *K*α radiation, λ = 0.71073 Å
Hall symbol: -P 2ybc	Cell parameters from 5377 reflections
*a* = 7.1134 (2) Å	θ = 2.6–33.0°
*b* = 10.9260 (3) Å	µ = 0.10 mm^−^^1^
*c* = 11.5291 (3) Å	*T* = 153 K
β = 98.485 (2)°	Prism, yellow
*V* = 886.25 (4) Å^3^	0.60 × 0.57 × 0.52 mm
*Z* = 4	

### Data collection

**Table:**

Bruker Kappa APEXII CCD diffractometer	1855 independent reflections
Radiation source: fine–focus sealed tube	1577 reflections with *I* > 2σ(*I*)
graphite	*R*_int_ = 0.018
φ and ω scans	θ_max_ = 26.6°, θ_min_ = 2.6°
Absorption correction: multi-scan (*SADABS*; Bruker, 2007)	*h* = −8→8
*T*_min_ = 0.659, *T*_max_ = 0.747	*k* = −13→13
9182 measured reflections	*l* = −14→14

### Refinement

**Table:**

Refinement on *F*^2^	Primary atom site location: structure-invariant direct methods
Least-squares matrix: full	Secondary atom site location: difference Fourier map
*R*[*F*^2^ > 2σ(*F*^2^)] = 0.041	Hydrogen site location: inferred from neighbouring sites
*wR*(*F*^2^) = 0.123	H-atom parameters constrained
*S* = 1.11	*w* = 1/[σ^2^(*F*_o_^2^) + (0.0664*P*)^2^ + 0.1717*P*] where *P* = (*F*_o_^2^ + 2*F*_c_^2^)/3
1855 reflections	(Δ/σ)_max_ < 0.001
132 parameters	Δρ_max_ = 0.30 e Å^−^^3^
0 restraints	Δρ_min_ = −0.21 e Å^−^^3^

### Special details

**Table:**

Geometry. All s.u.'s (except the s.u. in the dihedral angle between two l.s. planes) are estimated using the full covariance matrix. The cell s.u.'s are taken into account individually in the estimation of s.u.'s in distances, angles and torsion angles; correlations between s.u.'s in cell parameters are only used when they are defined by crystal symmetry. An approximate (isotropic) treatment of cell s.u.'s is used for estimating s.u.'s involving l.s. planes.
Refinement. Refinement of *F*^2^ against ALL reflections. The weighted *R*–factor *wR* and goodness of fit *S* are based on *F*^2^, conventional *R*–factors *R* are based on *F*, with *F* set to zero for negative *F*^2^. The threshold expression of *F*^2^ > 2σ(*F*^2^) is used only for calculating *R*–factors(gt) *etc*. and is not relevant to the choice of reflections for refinement. *R*–factors based on *F*^2^ are statistically about twice as large as those based on *F*, and *R*–factors based on ALL data will be even larger.

### Fractional atomic coordinates and isotropic or equivalent isotropic displacement parameters (Å^2^)

**Table:**

	*x*	*y*	*z*	*U*_iso_*/*U*_eq_	Occ. (<1)
O1	0.36987 (18)	0.74231 (10)	0.01694 (11)	0.0392 (4)	0.769 (3)
H1O	0.4139	0.7335	0.0881	0.059*	0.769 (3)
O1A	0.0897 (7)	1.0538 (5)	−0.2444 (3)	0.0555 (15)	0.231 (3)
H1OA	0.0636	1.1284	−0.2393	0.083*	0.231 (3)
O2	0.46630 (14)	0.80245 (8)	0.22874 (8)	0.0416 (3)	
O3	0.05289 (18)	1.25536 (12)	−0.17533 (10)	0.0643 (4)	
C1	0.30909 (15)	0.85929 (11)	−0.00259 (10)	0.0301 (3)	
H1A	0.3534	0.7780	0.0124	0.036*	0.231 (3)
C2	0.32180 (14)	0.94450 (10)	0.08990 (9)	0.0254 (3)	
C3	0.25975 (14)	1.06404 (10)	0.06490 (9)	0.0256 (3)	
H3	0.2690	1.1223	0.1267	0.031*	
C4	0.18496 (15)	1.09999 (11)	−0.04787 (10)	0.0301 (3)	
C5	0.17072 (16)	1.01185 (13)	−0.13782 (10)	0.0354 (3)	
H5	0.1177	1.0345	−0.2153	0.042*	0.769 (3)
C6	0.23194 (16)	0.89384 (13)	−0.11574 (10)	0.0361 (3)	
H6	0.2216	0.8358	−0.1777	0.043*	
C7	0.40012 (16)	0.90573 (11)	0.20988 (10)	0.0301 (3)	
C8	0.3997 (2)	0.99274 (13)	0.30966 (11)	0.0432 (3)	
H8A	0.4300	0.9484	0.3839	0.065*	
H8B	0.2738	1.0303	0.3053	0.065*	
H8C	0.4950	1.0567	0.3052	0.065*	
C9	0.11948 (17)	1.22703 (13)	−0.07529 (12)	0.0408 (3)	
C10	0.1355 (2)	1.31998 (13)	0.02060 (15)	0.0483 (4)	
H10A	0.0878	1.3989	−0.0117	0.073*	
H10B	0.2690	1.3285	0.0557	0.073*	
H10C	0.0603	1.2935	0.0807	0.073*	

### Atomic displacement parameters (Å^2^)

**Table:**

	*U*^11^	*U*^22^	*U*^33^	*U*^12^	*U*^13^	*U*^23^
O1	0.0458 (7)	0.0296 (6)	0.0432 (7)	0.0006 (5)	0.0100 (5)	−0.0058 (5)
O1A	0.056 (3)	0.080 (4)	0.028 (2)	−0.018 (3)	−0.0051 (18)	0.005 (2)
O2	0.0476 (6)	0.0300 (5)	0.0460 (5)	0.0046 (4)	0.0027 (4)	0.0099 (4)
O3	0.0627 (7)	0.0742 (8)	0.0516 (7)	0.0163 (6)	−0.0056 (5)	0.0302 (6)
C1	0.0222 (5)	0.0309 (6)	0.0383 (6)	−0.0045 (4)	0.0082 (4)	−0.0042 (5)
C2	0.0193 (5)	0.0284 (6)	0.0289 (6)	−0.0029 (4)	0.0046 (4)	0.0013 (4)
C3	0.0197 (5)	0.0283 (6)	0.0289 (6)	−0.0016 (4)	0.0034 (4)	0.0022 (4)
C4	0.0188 (5)	0.0392 (7)	0.0318 (6)	−0.0023 (5)	0.0022 (4)	0.0076 (5)
C5	0.0221 (6)	0.0562 (8)	0.0268 (6)	−0.0061 (5)	0.0002 (4)	0.0029 (5)
C6	0.0266 (6)	0.0491 (8)	0.0333 (6)	−0.0083 (5)	0.0062 (5)	−0.0102 (5)
C7	0.0271 (6)	0.0281 (6)	0.0352 (6)	−0.0018 (4)	0.0043 (4)	0.0065 (5)
C8	0.0586 (9)	0.0409 (7)	0.0285 (6)	0.0063 (6)	0.0012 (6)	0.0040 (5)
C9	0.0259 (6)	0.0486 (8)	0.0471 (7)	0.0029 (5)	0.0029 (5)	0.0203 (6)
C10	0.0455 (8)	0.0330 (7)	0.0667 (9)	0.0054 (6)	0.0090 (7)	0.0157 (6)

### Geometric parameters (Å, °)

**Table:**

O1—C1	1.3573 (16)	C4—C5	1.4078 (18)
O1—H1O	0.8400	C4—C9	1.4833 (18)
O1A—C5	1.357 (4)	C5—C6	1.373 (2)
O1A—H1OA	0.8400	C5—H5	0.9500
O2—C7	1.2297 (15)	C6—H6	0.9500
O3—C9	1.2204 (17)	C7—C8	1.4926 (17)
C1—C6	1.3908 (17)	C8—H8A	0.9800
C1—C2	1.4083 (16)	C8—H8B	0.9800
C1—H1A	0.9500	C8—H8C	0.9800
C2—C3	1.3951 (16)	C9—C10	1.493 (2)
C2—C7	1.4751 (15)	C10—H10A	0.9800
C3—C4	1.3870 (15)	C10—H10B	0.9800
C3—H3	0.9500	C10—H10C	0.9800
			
C1—O1—H1O	109.5	C5—C6—C1	119.96 (11)
C5—O1A—H1OA	109.5	C5—C6—H6	120.0
O1—C1—C6	118.89 (11)	C1—C6—H6	120.0
O1—C1—C2	120.91 (11)	O2—C7—C2	120.74 (11)
C6—C1—C2	120.20 (11)	O2—C7—C8	119.50 (11)
C6—C1—H1A	119.9	C2—C7—C8	119.76 (10)
C2—C1—H1A	119.9	C7—C8—H8A	109.5
C3—C2—C1	118.66 (10)	C7—C8—H8B	109.5
C3—C2—C7	121.78 (10)	H8A—C8—H8B	109.5
C1—C2—C7	119.56 (10)	C7—C8—H8C	109.5
C4—C3—C2	121.65 (11)	H8A—C8—H8C	109.5
C4—C3—H3	119.2	H8B—C8—H8C	109.5
C2—C3—H3	119.2	O3—C9—C4	120.31 (15)
C3—C4—C5	118.21 (11)	O3—C9—C10	120.26 (13)
C3—C4—C9	121.99 (12)	C4—C9—C10	119.43 (11)
C5—C4—C9	119.80 (11)	C9—C10—H10A	109.5
O1A—C5—C6	124.4 (3)	C9—C10—H10B	109.5
O1A—C5—C4	114.2 (3)	H10A—C10—H10B	109.5
C6—C5—C4	121.30 (11)	C9—C10—H10C	109.5
C6—C5—H5	119.3	H10A—C10—H10C	109.5
C4—C5—H5	119.3	H10B—C10—H10C	109.5
			
O1—C1—C2—C3	179.13 (11)	O1A—C5—C6—C1	−178.5 (3)
C6—C1—C2—C3	−1.47 (16)	C4—C5—C6—C1	0.28 (17)
O1—C1—C2—C7	−0.74 (16)	O1—C1—C6—C5	−179.54 (11)
C6—C1—C2—C7	178.67 (10)	C2—C1—C6—C5	1.04 (17)
C1—C2—C3—C4	0.59 (16)	C3—C2—C7—O2	−174.85 (10)
C7—C2—C3—C4	−179.55 (10)	C1—C2—C7—O2	5.01 (17)
C2—C3—C4—C5	0.69 (16)	C3—C2—C7—C8	4.99 (17)
C2—C3—C4—C9	−179.72 (10)	C1—C2—C7—C8	−175.15 (11)
C3—C4—C5—O1A	177.7 (2)	C3—C4—C9—O3	−179.39 (12)
C9—C4—C5—O1A	−1.9 (3)	C5—C4—C9—O3	0.20 (19)
C3—C4—C5—C6	−1.14 (17)	C3—C4—C9—C10	0.55 (18)
C9—C4—C5—C6	179.26 (11)	C5—C4—C9—C10	−179.86 (11)

### Hydrogen-bond geometry (Å, °)

**Table:**

*D*—H···*A*	*D*—H	H···*A*	*D*···*A*	*D*—H···*A*
O1—H1O···O2	0.84	1.78	2.5240 (15)	147.
O1A—H1OA···O3	0.84	1.58	2.369 (6)	156.
C6—H6···O3^i^	0.95	2.55	3.2797 (15)	134.

Symmetry codes: (i) −*x*, *y*−1/2, −*z*−1/2.
